# Influence of day and night temperature and carbon dioxide concentration on growth, yield, and quality of green butterhead and red oakleaf lettuce

**DOI:** 10.1371/journal.pone.0313884

**Published:** 2025-02-06

**Authors:** Sean T. Tarr, Roberto G. Lopez

**Affiliations:** Department of Horticulture, Michigan State University, East Lansing, MI, United States of America; Wageningen University, NETHERLANDS, KINGDOM OF THE

## Abstract

Production of lettuce (*Lactuca sativa*) within vertical farms is an expanding segment of controlled environment agriculture—precise manipulation of environmental parameters including mean daily temperature (MDT) and carbon dioxide (CO_2_) concentration enables year-round production, alongside color, yield, and crop size regulation. Our objectives included 1) quantify how MDT and CO_2_ interact to influence lettuce growth, development, and quality; 2) model lettuce growth under several MDTs and CO_2_ concentrations. Green butterhead ‘Rex’ and red oakleaf ‘Rouxaï RZ’ seedlings were transplanted into hydroponic tanks under a photosynthetic photon flux density of 300 μmol·m^‒2^·s^‒1^ for 17-h·d^‒1^. CO_2_ concentrations of 500, 800, or 1200 μmol·mol^−1^ and day/night and MDT setpoints of 22/15°C (MDT 20°C), 25/18°C (23°C), or 28/21°C (26°C) were maintained within growth chambers. ‘Rex’ fresh mass increased linearly with MDT, increasing by 18% from 20 to 26°C. ‘Rouxaï RZ’ fresh mass increased quadratically with MDT, with a 32% increase from 20 to 23°C, then a 7% increase from 23 to 26°C. Elevating CO_2_ concentrations from 500 to 800 μmol·mol^–1^ increased ‘Rouxaï RZ’ and ‘Rex’ fresh mass by 33 and 16%, respectively, while fresh mass did not increase from 800 to 1200 μmol·mol^–1^. Both cultivars had the greatest dry mass at 800 μmol·mol^–1^ CO_2_ across temperatures. At a high MDT, ‘Rouxaï RZ’ foliage color became more light, vibrant, and green, while a low MDT induced darker, grayer, and redder foliage. Tipburn occurred on ‘Rex’ across treatments, while 25% of ‘Rouxaï RZ’ were afflicted at 500 μmol·mol^−1^ CO_2_ and 67% at 1200 μmol·mol^−1^. At the light intensity studied, we recommend growing ‘Rex’ and ‘Rouxaï RZ’ at an 800 μmol·mol^−1^ CO_2_ concentration and MDT of 23°C for greatest biomass and leaf number, and slightly redder foliage in ‘Rouxaï RZ’ than at a 26°C MDT.

## Introduction

Lettuce (*Lactuca sativa*), as a global economic crop, plays a crucial role in the agricultural sector, notably in the United States, where it accounted for a staggering $3.5 billion in wholesale production in 2019 [1]. Although a significant proportion of leaf and romaine lettuce is field grown in California and Arizona, a rising demand for locally grown leafy greens has sparked interest in controlled environments (CE) production. From 2014 to 2019, sales of lettuce grown in CE increased by 28% due to benefits such as consistent production during off-seasons and stable pricing throughout the year [2, 3].

Vertical farms, warehouses, and shipping containers are among the common facilities used in CE food production. They enable the precise control of environmental conditions such as light duration, quantity, and quality; day and night temperatures; air flow; vapor pressure deficit (VPD); and carbon dioxide (CO_2_) concentration [3–5]. This precision, particularly in controlling factors such as mean daily temperature (MDT) and CO_2_ concentration, has a profound effect on plant growth, development, flavor, color, and nutrient content. These environmental control capabilities combined with lettuce’s compact growth, short production time, and high market demand make it an ideal candidate for CE production, potentially improving harvest frequency and space efficiency [5, 6].

Temperature plays a pivotal role in plant growth; the rate of development of lettuce increases linearly with increasing temperature, from a species-specific base temperature (T_b_) up to an optimum temperature (T_opt_), after which the developmental rate declines [7]. Excessive temperatures late in the growing cycle can induce bolting in lettuce, reducing overall quality as heads become loose, leaves become bitter, and tipburn risk increases. Additionally, temperature can influence crop quality aspects such color in red-leaf lettuce cultivars. Like many plants, lettuce uses C_3_ carbon fixation, which is responsive to atmospheric CO_2_ concentrations. Elevating CO_2_ availability decreases photorespiration, raising the photosynthetic rate and light saturation point, thus increasing growth until a species-specific CO_2_ concentration saturation point is reached [8, 9]. There is an initial high impact on growth as CO_2_ is elevated above ambient levels (~420 μmol·mol^‒1^) but benefits diminish as the saturation point is approached [8, 9]. CO_2_ and MDT interact to influence growth and development—at greater CO_2_ concentrations, the T_opt_ for photosynthesis typically increases [9, 10]. Beyond the T_opt_, the rate of carboxylation to oxygenation decreases, reducing photosynthetic efficiency due to photorespiration [10].

The impact MDT has on the growth and development of lettuce has been investigated previously [5, 11, 12]. For example, Ouyang et al. (2020) grew ‘Grand Rapids TBR’ at 16, 18, and 20°C under a continuous photosynthetic photon flux density (*PPFD*) of 210 μmol∙m^–2^∙s^–1^ for 30 days after transplant [12]. Lettuce shoot fresh mass (SFM) and height was 38 and 18% (9.9 g and 1.9 cm) greater at 20°C than at 16°C and shoot dry mass (SDM) was 14% (0.5 g) greater at 18 or 20°C than at 16°C [12].

The influence of CO_2_ concentration on lettuce growth and development in CEs has also been investigated previously [13–16]. SFM of lettuce ‘Blonde of Paris Batavia’ and ‘Oak Leaf’ increased by 55 and 77% (46 and 34 g), respectively, when the CO_2_ concentration was raised from 400 to 700 μmol·mol^–1^ under a *PPFD* of 400 μmol∙m^–2^∙s^–1^ at day/night (14 h/10 h) temperatures of 25/18°C [16]. Additionally, the photosynthetic rate, apparent quantum yield, antioxidant capacity, and water-use efficiency increased for both cultivars [16].

Given the strong influence of MDT and CO_2_ concentration on lettuce growth, development, and quality, identifying the environmental parameters for improved resource-use efficiency and yield in CEs is needed. The objectives of this study were to 1) quantify how MDT and CO_2_ concentration influence lettuce growth, development, quality, and yield; and 2) create models for predicting growth and development under various MDTs and CO_2_ concentrations. We hypothesized that increasing CO_2_ concentration would increase biomass production of lettuce across all temperatures, but there would be less of an effect shifting from the moderate to the highest tested CO_2_ concentration compared to the shift from the low to medium CO_2_ concentrations.

## Materials and methods

### Plant material and propagation conditions

On 28 Apr. 2020, 09 June 2020, 27 July 2020, 16 Sept. 2020, 12 Nov. 2020, and 07 Jan. 2021, and 20 Feb. 2021, seeds of red oakleaf lettuce ‘Rouxaï RZ’ and green butterhead lettuce ‘Rex’ (Rijk Zwaan; Salinas, CA, USA) were sown into 200-cell (2.5 cm × 2.5 cm) rockwool plugs (AO 25/40 Starter Plugs; Gordan, Milton, ON, Canada). The cultivars were selected due to their use in previous indoor production studies and commercial relevance. Plugs were placed in trays and presoaked in deionized water with a pH of 4.4 to 4.5 adjusted using diluted (1:31) 95 to 98% sulfuric acid (J.Y. Baker, Inc.; Phillipsburg, NJ, USA). The trays were covered with translucent plastic domes for 3 d to maintain high humidity during germination. Trays were placed in a 2.4-m-wide, 4.1-m-long, 2.4-m-tall walk-in growth chamber (Hotpack environmental room UWP 2614–3; SP Scientific, Warminster, PA, USA) with an MDT of 22°C, CO_2_ concentration of 500 μmol·mol^‒1^, and relative humidity of 60%. Light-emitting diode fixtures (Ray66 Indoor PhysioSpec; Fluence Bioengineering, Austin, TX, USA) provided a total photon flux density (400–800 nm) of 180 μmol∙m^–2^∙s^–1^ and a light ratio (%) of 19:39:39:3 blue (400–500 nm): green (500–600 nm): red (600–700 nm): far-red (700–800 nm) radiation for 24 h. After 3 d, the photoperiod was reduced to 20 h until transplant at 11 d. Seedlings were sub-irrigated with deionized water supplemented with water-soluble fertilizer providing (in mg∙L^–1^): 125 N, 18 P, 138 K, 73 Ca, 47 Mg, 1.56 Fe, 0.52 Mn, 0.36 Zn, 0.21 B, 0.21 Cu, 35 S, and 0.01 Mo (12N–1.8P–13.3K RO Hydro FeED; JR Peters, Inc., Allentown, PA, USA). The pH and electrical conductivity (EC) were adjusted to 5.6 and 1.6 dS·m^–1^, respectively, as determined with a pH/EC probe (HI 991, 301 pH/TDS/ Temperature Monitor; Hanna Instruments, Smithfield, RI, USA). The pH was adjusted using potassium bicarbonate and sulfuric acid, while the EC was adjusted by adding deionized water and concentrated nutrient solution.

### Hydroponic systems

Eleven days after sowing, 14 seedlings of each cultivar were transplanted 20-cm-apart into six 250 L, 0.9-m-wide by 1.8-m-long deep-flow hydroponic systems (Active Aqua premium high-rise flood table; Hydrofarm, Petaluma, CA, USA) distributed within walk-in growth chambers described previously. Each hydroponic system contained a 4-cm-thick extruded polystyrene foam sheet to float on the nutrient solution. Plastic net baskets were placed into 4-cm-diameter holes in the polystyrene foam, and seedlings were placed in the baskets, so the rockwool was in contact with the nutrient solution. Deionized water supplemented with water-soluble fertilizer providing (in mg·L^–1^) 150 N, 22 P, 166 K, 87 Ca, 25 Mg, 1.9 Fe, 0.62 Mn, 0.44 Zn, 0.25 B, 0.25 Cu, and 0.01 Mo (12N–1.8P–13.3K RO Hydro FeED; JR Peters, Inc.), and 0.31 g·L^–1^ magnesium sulfate (Pennington Epsom salt; Madison, GA, USA). The EC and pH were adjusted daily to maintain an EC of 1.7 dS·m^–1^ and pH of 5.6, as described previously. Air pumps (Active Aqua 70 L·min^–1^ commercial air pump; Hydrofarm) connected to air stones (Active Aqua air stone round 10.2 cm × 2.5 cm; Hydrofarm) were used to increase the dissolved oxygen concentration.

### Growth chamber environmental conditions

The air day/night (17 h/7 h) and MDT set points in each growth chamber were 22/15 (20°C), 25/18 (23°C), or 28/21 (26°C), measured every 5 s by a resistance temperature detector (Platinum RTD RBBJL-GW05A-00-M 36B; SensorTec, Inc., Fort Wayne, IN, USA) and logged by a C6 controller (Environmental Growth Chambers, Chagrin Falls, OH, USA). CO_2_ was maintained at 500, 800, or 1200 μmol·mol^–1^ in each chamber with compressed CO_2_ injection, measured with a CO_2_ sensor (GM86P; Vaisala, Helsinki, Finland) and logged by a C6 Controller (Environmental Growth Chambers) every 5 s. A positive fixed difference between air and night temperatures (DIF) of 7°C was used for each temperature treatment. Relative humidity had a target setpoint of 60%. *PPFD*s of 300 μmol∙m –2∙s^–1^ were provided for 17 h∙d^–1^ by LED fixtures (Ray66; Fluence Bioengineering), providing a DLI of 18.4 mol∙m –2∙d^–1^, averaged over several measurements (Table 1). The LEDs were mounted 95 cm above the crop canopy. Every 30 s, water temperature, leaf temperature, and *PPFD* were measured using a thermistor (ST-100; Apogee Instruments, Logan, UT, USA), infrared thermocouple (OS36-01-T-80F; Omega Engineering, INC. Norwalk, CT, USA), and quantum sensor (LI-190R; LI-COR Biosciences, Lincoln, NE, USA), respectively, with means logged every hour by a CR-1000 datalogger (Campbell Scientific, Logan, UT, USA). The average horizontal air speed was approximately 0.37 m⋅s^−1^ over the growing canopy measured with an anemometer (HHF803; Omega Engineering, Norwalk, CN, USA). Vertical airflow and inter-canopy airflow was not provided or assessed.

**Table 1:** Mean (± SD) day and night air, canopy, and water temperatures; carbon dioxide (CO_2_) concentrations; photosynthetic photon flux density (PPFD); and vapor pressure deficit (VPD) during 36 or 37 days of indoor deep flow hydroponic production for butterhead lettuce ‘Rex’ and red oakleaf lettuce ‘Rouxaï RZ’, respectively.

Temperature (°C)				CO_2_ (μmol∙mol^‒1^)			*PPFD* (μmol∙m^‒2^∙s^‒1^)			VPD (kPa)					
Air day	Air night	Canopy	Water							Day			Night		
22.0 ± 0.1	15.3 ± 0.1	22.2 ± 3.3	20.6 ± 1.0	499.2	±	44.9	307.8	±	5.8	1.08	±	0.05	0.67	±	0.03
22.0 ± 0.2	15.4 ± 0.3	23.1 ± 3.2	21.3 ± 3.2	801.2	±	12.3	303.4	±	7.7	1.11	±	0.08	0.69	±	0.08
21.9 ± 0.1	15.1 ± 0.0	22.3 ± 3.7	20.5 ± 1.0	1173.2	±	187.9	295.6	±	7.0	1.00	±	0.35	0.51	±	0.25
24.9 ± 0.4	18.3 ± 0.2	25.7 ± 3.7	23.9 ± 1.0	503.1	±	51.4	299.9	±	9.3	1.22	±	0.06	0.81	±	0.04
25.4 ± 1.3	18.5 ± 0.8	24.8 ± 3.6	23.9 ± 2.7	798.3	±	23.0	300.0	±	5.7	1.34	±	0.05	0.90	±	0.06
25.0 ± 0.0	18.2 ± 0.0	25.5 ± 3.1	23.3 ± 2.0	1166.1	±	204.5	295.6	±	8.9	1.16	±	0.23	0.70	±	0.08
28.1 ± 0.5	21.3 ± 0.4	28.0 ± 3.1	26.4 ± 1.1	497.8	±	58.4	299.4	±	10.2	1.73	±	0.20	1.17	±	0.13
27.9 ± 0.1	21.2 ± 0.0	27.8 ± 3.4	26.3 ± 2.5	801.5	±	15.3	297.9	±	12.5	1.64	±	0.03	1.09	±	0.01
27.9 ± 0.1	21.1 ± 0.0	27.5 ± 3.4	26.2 ± 1.2	1167.8	±	205.1	299.4	±	5.8	1.25	±	0.25	0.63	±	0.06

### Growth data collection and analysis

Parameters assessed for lettuce quality included the foliage coloration of ‘Rouxaï RZ’, relative chlorophyll concentration (RCC), the maximum photosystem II quantum yields (F_v_/F_m_), and the dry mass. The foliage coloration of ten ‘Rouxaï RZ’ plants in each treatment was measured 35 d after sowing with a tristimulus colorimeter (Chroma Meter CR-400; Konica Minolta Sensing, Inc., Chiyoda, Tokyo), reported as International Commission on Illumination (CIE) L*a*b* color space values, which were then converted to hue angle (h°) and chroma (C*). The RCC of the most recent fully expanded leaf of ten plants of each cultivar in each treatment was then estimated with a SPAD meter (MC-100 Chlorophyll Meter; Apogee Instruments, Logan, UT, USA). One leaf of ten plants per treatment was then dark acclimated for >15 min using three of the manufacturer-supplied clips and then exposed to 3500 μmol·m^–2^·s^–1^ of red radiation (peak wavelength 650 nm) to saturate photosystem II and the fluorescence was measured, averaged, and reported as F_v_/F_m_ by a portable chlorophyll fluorescence meter (Handy Plant Efficiency Analyzer; Hansatech Instruments Ltd., Norfolk, UK).

‘Rouxaï RZ’ and ‘Rex’ were harvested 36 and 37 d after sowing, respectively. SFM (g), length and width (cm) of the sixth fully expanded leaf, and leaf number (when >5 cm) were measured on ten plants of each cultivar per treatment. Plant height from the roots to the highest point of the foliage and the foliage diameter at the widest point and perpendicular to the widest point was measured with a ruler and recorded. Incidence, but not severity, of tipburn was recorded. Plants were observed for bolting and no bolting was observed. To provide an integrated measurement of plant size, the growth index (GI) was calculated using the crop height and two foliage diameters mentioned previously (GI = {plant height + [(diameter 1 + diameter 2)/2]}/2). The plant material was placed in a forced-air drier maintained at 75°C for at least 3 d, weighed, and the SDM was recorded.

The study was conducted using a randomized block design, where the blocks were defined by sequential time periods. Each block corresponded to a two-month cycle, resulting in six blocks (T1 through T6). Within each block, a single CO_2_ treatment (500, 800, or 1200 μmol·mol^–1^) was applied to all three growth chambers. The CO_2_ treatments were assigned to blocks in the following sequence: 500 μmol·mol^–1^ in T1 and T2, 800 μmol·mol^–1^ in T3 and T4, and 1200 μmol·mol^–1^ in T5 and T6. Within each CO_2_ treatment block, all three temperature treatments were randomized across the chambers. This randomization was replicated, resulting in each CO_2_ treatment being paired with each temperature treatment twice before advancing to the next CO_2_ level. The temperature treatments were distributed between each harvest within each CO_2_ block to ensure randomization.

This design allowed for the examination of the interaction between CO_2_ concentration and temperature on lettuce growth, with each growth chamber serving as the experimental unit for the temperature treatments within the given CO_2_ treatment block. Data were collected from 10 randomly selected lettuce plants in each growth chamber at the end of each cycle, providing a total of 20 plants sampled per treatment combination per time period.

Data were analyzed separately by cultivar with SAS (version 9.4; SAS Institute, Cary, NC, USA) mixed model procedure (PROC MIXED) for analysis of variance (ANOVA), tests of normality and homogeneity of variances were performed, and pairwise comparisons were performed with Tukey-Kramer difference test (*p* ≤ 0.05). SigmaPlot (version 14.5, Systat Software, Inc., San Jose, CA, USA) was used for regression analysis.

## Results

### Shoot fresh and dry mass

**Fig 1:**
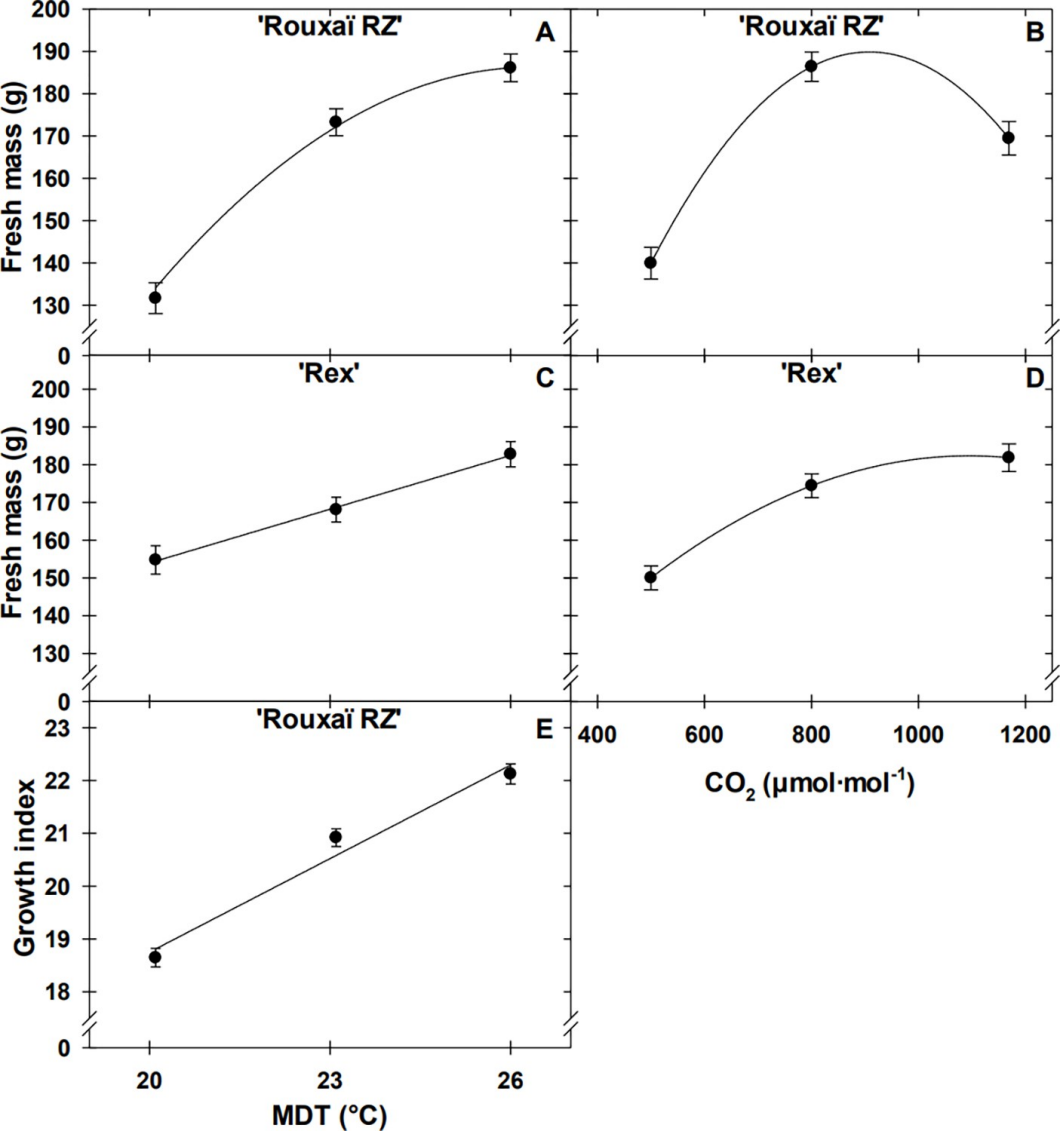
Effects of mean daily temperature (MDT; 20, 23, and 26°C) and carbon dioxide (CO_2_ concentration; 500, 800, 1200 μmol·mol^–1^) on red oakleaf lettuce ‘Rouxaï RZ’ fresh mass (A and B) and growth index (E), and green butterhead lettuce ‘Rex’ fresh mass (C and D). Model predictions are represented by lines; coefficients are in Table 3; error bars represent standard errors.

The SFM of ‘Rouxaï RZ’ increased quadratically with MDT; from 20 to 23°C, SFM increased by 32% (41.6 g), then 7% (12.9 g) from 23 to 26°C (Tables 2 and 3; Fig 1A). ‘Rex’ SFM increased linearly by 18% (28.0 g) from 20 to 26°C (Tables 2 and 3; Fig 1C). Both cultivars showed quadratic increases in SFM as CO_2_ concentration increased (Table 2). Elevating CO_2_ from 500 to 800 μmol·mol^–1^ resulted in SFM increasing by 33 and 16% (46.5 and 24.4 g) for ‘Rouxaï RZ’ and ‘Rex’, respectively, without additional biomass accumulation as CO_2_ increased from 800 to 1200 μmol·mol^–1^ (Tables 2 and 3; Fig 1B, 1D).

**Table 2:** Influence of mean daily temperature (MDT;°C) and carbon dioxide (CO_2_; μmol·mol^–1^) on height and width (cm); growth index; leaf length and width (cm) and number; shoot fresh and dry mass (g); hue angle (h°); chroma (C*); International Commission on Illumination (CIE) L* color value of red oakleaf lettuce ‘Rouxaï RZ’ and green butterhead lettuce ‘Rex’. Data represent the mean of two replications and cultivars with 10 samples. Analyses of variance for the effects of MDT and CO_2_ and their interaction are included below each cultivar mean. Within-column means of a given treatment with different letters were significantly different according to Tukey’s honestly significant difference test (P < 0.05).

MDT	CO_2_	Height	Width	Growth index	Leaf length	Leaf width	Leaf (no.)	Fresh mass	Dry mass	h°	C*	L*
‘Rouxaï RZ’												
20		12.5 c	24.8 c	18.6 c				131.6 c		71.4 b	6.6 b	29.9 b
23		14.3 b	27.5 b	20.9 b				173.3 b		77.5 b	7.8 ab	31.2 ab
26		15.4 a	28.7 a	22.1 a				186.1 a		89.2 a	9.1 a	32.5 a
	500	^z^						139.9 b				
	800							186.4 a				
	1200							169.5 ab				
20	500				12.3 c	18.9 b	20.2 de		4.80 d			
	800				14.3 ab	20.8 ab	24.8 cde		6.70 ab			
	1200				13.4 bc	20.5 ab	19.5 e		5.25 cd			
23	500				14.3 ab	20.4 a	28.0 abc		6.06 abc			
	800				14.4 ab	20.4 ab	31.5 ab		7.31 ab			
	1200				14.2 abc	20.8 ab	27.7 bd		6.24 b			
26	500				14.9 ab	20.9 a	28.5 abc		6.42 abc			
	800				15.0 ab	21.0 ab	33.4 ab		7.26 ab			
	1200				15.0 a	21.8 a	31.3 ac		7.39 a			
MDT		***^y^	***	***	***	***	***	***	***	***	***	***
CO_2_		ns	ns	ns	ns	ns	ns	*	ns	ns	ns	ns
MDT×CO_2_		ns	ns	ns	***	*	**	ns	***	ns	ns	ns
‘Rex’												
20		12.2 b				15.1 a		154.8 c		–^x^	–	–
23		13.1 a				15.0 ab		168.1 b		–	–	–
26		12.8 a				14.6 b		182.8 a		–	–	–
	500							150.0 b		–	–	–
	800							174.4 a		–	–	–
	1200							181.9 a		–	–	–
20	500		19.0 e	15.4 e	11.7 c		21.5 eg		5.68 c	–	–	–
	800		21.7 cd	16.9 d	12.0 abc		24.5 def		7.63 ab	–	–	–
	1200		21.6 cd	17.1 cd	11.6 bc		23.5 fg		6.69 bc	–	–	–
23	500		21.4 d	17.3 bcd	12.6 ab		28.0 cf		6.94 b	–	–	–
	800		21.8 cd	17.5 bcd	12.4 abc		27.8 def		7.68 ab	–	–	–
	1200		23.2 ab	18.1 ab	11.9 abc		28.5 bcde		6.96 b	–	–	–
26	500		22.8 abc	17.7 abcd	12.9 a		32.3 bd		7.26 b	–	–	–
	800		22.6 bcd	18.0 abc	11.8 bc		35.0 bc		8.35 a	–	–	–
	1200		24.1 a	18.5 a	11.6 bc		43.1 a		7.48 ab	–	–	–
MDT		***	***	***	**	*	***	***	***	–	–	–
CO_2_		ns	*	*	ns	ns	ns	*	*	–	–	–
MDT×CO_2_		ns	***	*	***	ns	***	ns	*	–	–	–

^z^ blank cells were not significant

^y^ NS, *, **, *** represent non-significant or significant difference at P ≤ 0.05, 0.01, and 0.001, respectively.

^x^ Data not collected.

**Table 3:** Equations of regression analysis and r^2^ or R^2^ for mean shoot fresh and dry mass (g), growth index, leaf number, and leaf length (cm) in response to mean daily temperature (MDT; 20, 23, and 26°C) and carbon dioxide (CO_2_ concentration; 500, 800, 1200 μmol·mol^–1^) of green butterhead lettuce ‘Rex’ and red oakleaf lettuce ‘Rouxaï RZ’. All models are in the form of: ƒ = y0 + a*MDT + b*CO_2_ + c*MDT^2^ + d*CO_2_^2^ + e*MDT*CO_2_.

Parameter	y0	(a) MDT	(b) CO_2_	(c) MDT^2^	(d) CO_2_^2^	(e) MDT*CO_2_	R^2^ or r^2^
‘Rex’							
Fresh mass (g)	6.23E1^z^	4.61	^y^				0.15
	1.90E1^x^	8.19E-1					
Fresh mass (g)	7.18E1		2.03E-1		-9.31E-5		0.21
	2.40E1		6.20E-2		3.68E-5		
Dry mass (g)	-3.13	1.68E-1	1.60E-2		-9.49E-6		0.38
	9.98E-1	2.60E-2	2.00E-3		1.23E-6		
Growth index	9.71	2.73E-1	2.00E-3				0.42
	7.11E-1	2.90E-2	2.58E-4				
Leaf (no.)	1.37E2	-1.04E1	-4.50E-2	2.33E-1		2.00E-3	0.58
	4.88E1	4.16	1.30E-2	8.90E-2		5.74E-4	
‘Rouxaï RZ’							
Fresh mass (g)	-9.15E2	8.54E1		-1.66			0.46
	2.37E2	2.08E1		4.51E-1			
Fresh mass (g)	-6.05E1		5.53E-1		-3.05E-4		0.31
	2.73E1		7.10E-2		4.20E-5		
Dry mass (g)	-5.86	2.42E-1	1.70E-2		-9.60E-6		0.55
	8.46E-1	2.20E-2	2.00E-3		1.05E-6		
Growth Index	7.17	5.81E-1					0.51
	9.89E-1	4.30E-2					
Leaf (no.)	-1.45E2	1.25E1	3.00E-2	-2.55E-1	-3.15E-5	1.00E-3	0.59
	3.73E1	3.18	1.40E-2	6.80E-2	5.47E-6	4.20E-4	
Leaf length (cm)	4.47	2.69E-1	9.00E-3		-4.89E-6		0.30
	1.27	3.40E-2	3.00E-3		1.59E-6		

^z^ Coefficients for model equations were used to generate Figs 1 and 2

^y^ Blank cells = 0

^x^ Standard error (se)

**Fig 2:**
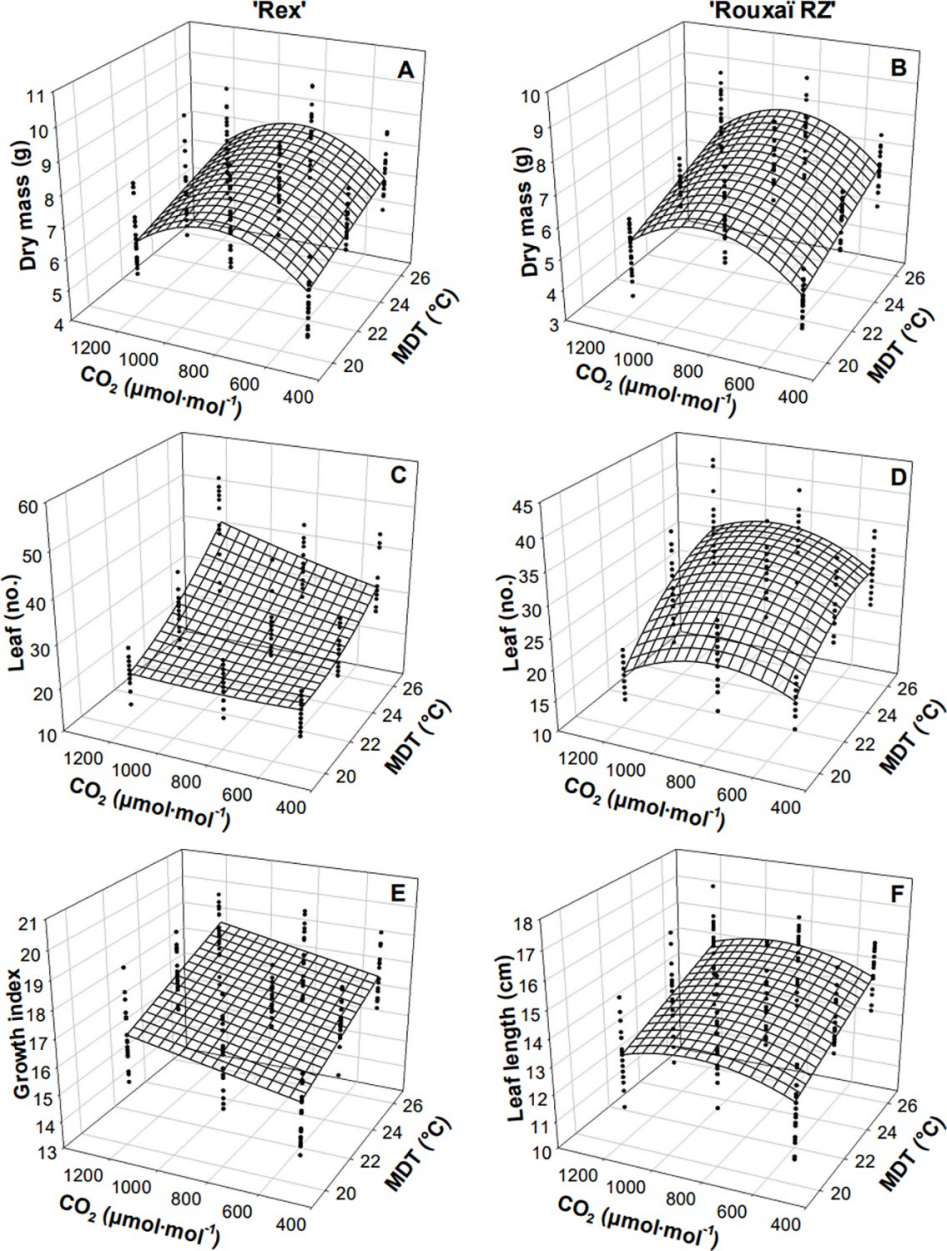
Effects of carbon dioxide concentration (CO_2_; 500, 800, 1200 μmol·mol^–1^) and mean daily temperature (MDT; 20, 23, and 26°C) on green butterhead lettuce (*Lactuca sativa*) ‘Rex’ shoot dry mass (A), leaf number (C), and growth index (E) and red oakleaf lettuce ‘Rouxaï RZ’ shoot dry mass (B), leaf number (D), and leaf width (F). Model predictions are represented by response surfaces; coefficients are in Table 3.

MDT and CO_2_ concentration interacted to influence SDM of both cultivars (Tables 2 and 3; Fig 2A and 2B). Regardless of MDT, the greatest SDM (~7.1 and ~7.9 g for ‘Rouxaï RZ’ and ‘Rex’, respectively) was recorded at a CO_2_ concentration of 800 μmol·mol^–1^, while the lowest was at 20°C and a CO_2_ concentration of 500 or 1200 μmol·mol^–1^ (5.0 and 6.2 g for ‘Rouxaï RZ’ and ‘Rex’, respectively). ‘Rex’ SDM increased from 20 to 23°C at 500 and 1200 μmol·mol^–1^ CO_2_.

### Morphology

‘Rouxaï RZ’ GI increased linearly with MDT, while the GI of ‘Rex’ was influenced by an interaction of CO_2_ and MDT (Tables 2 and 3; Figs 1E and 2E). ‘Rouxaï RZ’ GI increased by 19% as MDT increased from 20 to 26°C. GI of ‘Rex’ increased by 20% as MDT and CO_2_ concentration increased from 20°C and 500 μmol·mol –1 CO_2_ to 23°C and 1200 μmol·mol ^‒ 1^ CO_2_, respectively, or an MDT of 26°C.

Leaf number was influenced by the interaction of MDT and CO_2_ concentration for both cultivars (Tables 2 and 3; Fig 2C and 2D). The leaf number of ‘Rex’ was primarily influenced by the MDT, and as MDT increased from 20 to 23°C and 23 to 26°C, an average of 5 and 8 more leaves unfolded, respectively. The fewest number of leaves (22 leaves) was observed for ‘Rex’ at a CO_2_ concentration of 500 or 1200 μmol·mol^‒1^ at an MDT of 20°C, while the greatest number of leaves (43) was at a CO_2_ concentration of 1200 μmol·mol^‒1^ and MDT of 26°C. However, ‘Rouxaï RZ’ unfolded 21 leaves at an MDT of 20°C and an additional 8 leaves at 23°C, regardless of CO_2_ concentration. Only 4 additional leaves unfolded as the MDT increased from 23 to 26°C at a CO_2_ concentration of 1200 μmol·mol^‒1^.

Leaf length of both cultivars and leaf width of ‘Rouxaï RZ’ were influenced by the interaction of MDT and CO_2_, while leaf width of ‘Rex’ was influenced only by MDT (Tables 2 and 3; Fig 2F). Leaves of ‘Rouxaï RZ’ were 22% (2.7 cm) longer and 15% (2.9 cm) wider at an MDT of 26°C and CO_2_ concentration of 1200 μmol·mol^‒1^ than those grown at 20°C and 500 μmol·mol^‒1^ CO_2_. At 500 μmol·mol^‒1^ CO_2_, ‘Rex’ leaves were 10% longer at an MDT of 26°C than at 20°C, and were 4% wider at 20°C than at 26°C.

Tipburn incidence, recorded as the percentage of plants affected, was 25% and 67% at CO_2_ concentrations of 500 and 1200 μmol·mol^‒1^ for ‘Rouxaï RZ’. Tipburn was present on all of ‘Rex’ irrespective of MDT or CO_2_ concentration.

### F_v_/F_m_, relative chlorophyll concentration, and pigmentation

The maximum photosystem II quantum yields (F_v_/F_m_) ranged from 0.813 to 0.861, suggesting minimal impacts of stress on photosynthetic reactions. For ‘Rex’, the relative chlorophyll concentrations were 7% lower at 23°C (30.6) than at 20 or 26°C (32.6)

Foliage pigmentation of ‘Rouxaï RZ’ was influenced by MDT (Table 2). As MDT increased from 20 to 26°C, h° increased from 71.4 to 89.2, C* from 6.6 to 9.1, and L* from 29.9 to 32.5. These greater h°, C*, and L* values correspond to slightly lighter and more vibrant green foliage than the darker-gray, more yellow/red foliage with lower values.

### Discussion

Lettuce sales and yield are primarily determined by marketable fresh mass, with time to harvest and quality parameters such as color, plant size, and tipburn incidence being of particular importance to growers. Lettuce biomass accumulation varies by cultivar [17–19] and depends on stage of growth and time to harvest [20, 21], and environmental conditions, including *PPFD* [17, 19, 22], MDT [17, 20, 23], and CO_2_ concentration [13, 21, 24]. In the present study, SFM of ‘Rouxaï RZ’ and ‘Rex’ were influenced by CO_2_ and MDT independently, while SDM was influenced by the interaction of CO_2_ and MDT. SFM of both cultivars increased as MDT, with a 6°C positive difference in day and night temperature, increased from 20 to 26°C, with ‘Rex’ and ‘Rouxaï RZ’ following linear and quadratic responses, respectively (Table 2; Figs 1A, 1C; 2A and 2B). The quadratic response of ‘Rouxaï RZ’, with reduced SFM gain from the 23 to 26°C MDTs, may indicate the optimal temperature was being reached, after which a plateau and decline will occur until growth ceases and the maximum temperature is reached. Given this reduced SFM benefit at the higher MDT, growers should consider if the yield increase at 26°C is worthwhile compared to the energy cost of maintaining the higher temperature. Overall, the temperature response we saw aligns with other studies observing increased growth rate and biomass accumulation under heightened MDTs in lettuce [5, 20]. The recommended MDT for lettuce varies by variety and cultivar, but Choi et al. (2000) suggested a 22–26°C day and 15–20°C night temperature during the early and middle growth stages, followed by a 20–24°C and 15–20°C during the later stages of growth for butterhead ‘Omega’ and leaf lettuce ‘Grand Rapids’. In that study, the rate of photosynthesis and transpiration 15, 25, and 35 days after treatment of day/night temperatures of 10/7, 20/15, and 30/25°C, the high temperature treatment of 30/25°C had the greatest photosynthetic and transpiration rates at 25 days, while 20/15°C had the greatest at 35 days after treatment [20]. Altering the temperature based upon growth stages may provide opportunities for further optimization within plant factories if divisions within the facilities allow for temperature control between planting dates of crops.

In the present study, SFM of both cultivars only increased as the CO_2_ concentration was raised from 500 to 800 μmol·mol^–1^, with no further increases from 800 to 1200 μmol·mol^–1^ (Table 2). Caplan (2018) reported a similar response: butterhead lettuce ‘Fairly’ grown at an MDT of 22°C under *PPFD*s ranging from 156 to 330 μmol·m^−2^·s^−1^ and CO_2_ concentrations from 400 to 1300 μmol·mol^–1^ had the greatest SFM and SDM at 850 μmol·mol^−1^ CO_2_, while yields decreased as CO_2_ concentration increased [25]. Conversely, the SFM of lettuce ‘Partavousi’ increased by 6 and 55% as CO_2_ concentration increased from 400 to 800 and 800 to 1200 μmol·mol^−1^, respectively, under a *PPFD* of 300 μmol·m^−2^·s^−1^ and at an MDT of 25°C for 40 d, without additional biomass accumulation at CO_2_ concentrations of 1200 to 1600 μmol·mol^−1^ [21]. The reduced impact on growth at higher CO_2_ concentrations in the present study may be due to the CO_2_ saturation point being reached for the tested cultivars, after this saturation point the photosynthetic rate would no longer be limited by CO2 availability, but instead factors such as light, nutrients, or enzyme capacity. Additionally, there may have been other factors limiting lettuce growth once CO_2_ was elevated, such as the onset of tipburn due to the lack of vertical or inter-canopy airflow while under rapid growth conditions.

SDM was influenced by the interaction of CO_2_ and MDT for both cultivars (Tables 2 and 3; Fig 2A and 2B). Regardless of MDT, the greatest SDM occurred at a CO_2_ concentration of 800 μmol·mol^−1^, while at higher or lower CO_2_ concentrations, SDM was primarily influenced by MDT. Esmaili et al. observed that the SDM of ‘Partavousi’ increased by 31 and 147% as CO_2_ concentration increased from 400 to 800 and 800 to 1200 μmol·mol^−1^, respectively, with similar SDM at 1200 and 1600 μmol·mol^−1^ [21]. Within the conditions of our study, the 800 μmol·mol^−1^ CO_2_ concentration appears to be optimal for biomass accumulation for both SFM and SDM across temperature treatments.

The GI, a measure of plant size that integrates plant height and width, was greatest at an MDT of 26°C for both cultivars, while ‘Rex’ was marginally influenced by the interaction of CO_2_ and MDT (Tables 2 and 3; Figs 1E and 2E). Tarr et al. reported MDT only influencing the GI of ‘Rouxaï RZ’ as it increased from 20 to 23°C [17]. The size of plants can impact recommended planting density and packaging of heads of lettuce into clamshells; understanding that size increases within MDTs of 20 to 26°C enables growers to adjust conditions based upon market preferences. Leaf size was influenced by the interaction of MDT and CO_2_, but primarily by MDT (Table 2). MDT has been suggested to have a greater impact on leaf mass area (leaf dry mass per leaf area) than CO_2_ concentration [26]. Both cultivars had the shortest leaves at 20°C, while ‘Rouxaï RZ’ leaves were narrowest and ‘Rex’ leaves were widest at 20°C.

The interaction of CO_2_ concentration and MDT impacted the leaf unfolding rate for both cultivars (Tables 2 and 3; Fig 2C and 2D). Leaf unfolding increased from 20 to 26°C when CO_2_ concentrations were pooled, as expected with developmental rates increasing up to the T_opt_ [27]. The influence of CO_2_ and MDT on the leaf unfolding rate of lettuce is not well documented. Lettuce ‘Grand Rapids’ grown at an MDT of 16.7°C and ~500 μmol·mol^−1^ CO_2_ unfolded 3 more leaves than at 18.3°C and at CO_2_ concentrations of 200–400 μmol·mol^−1^ [14]. In CO_2_-limited conditions, the leaf unfolding rate and photosynthetic rate may be restricted as photorespiration occurs [8].

A major concern for CE lettuce producers is tipburn, the necrosis on a leaf margin induced by calcium deficiency [5, 22]. Lettuce undergoing rapid growth with limited transpiration at the growing point is susceptible to tipburn. In the current study, tipburn incidence of ‘Rouxaï RZ’ was greatest under a PPFD of 300 μmol∙m^‒2^∙s^‒1^ at a CO_2_ concentration of 1200 μmol·mol^−1^ (Table 2), while all ‘Rex’ treatments had signs of tipburn. We did not assess severity of tipburn, so distinctions between beginning signs of tipburn development on inner leaves at harvest and more severe tipburn occurrence was not made. When grown at an MDT of 22°C and under a *PPFD* of 330 μmol·m^−2^·s^−1^, tipburn occurrence in butterhead lettuce ‘Fairly’ was not observed at CO_2_ concentrations of 400 and 550 μmol·mol^−1^, but 10, 10, 25, and 33% of plants had tipburn at 700, 850, 1000, and 1300 μmol·mol^−1^ CO_2_, respectively [25]. The increased incidence of tipburn at elevated CO_2_ concentrations is likely due to a reduction in stomatal conductance [8, 25] as stomata close in elevated CO_2_ concentrations, reducing transpiration and, consequently, calcium movement to the growing point. MDT did not influence tipburn occurrence, aligning with Tarr et al. [17]. There are confounding reports in the literature about the influence of MDT on lettuce tipburn, as results varied by cultivar and environmental conditions [23, 28, 29]. The VPD may have influenced tipburn incidence in our study. We maintained a constant 60% relative humidity at 20, 23, and 26°C, which translates to VPDs of ~0.9, 1.1, and 1.3 kPa, respectively (Table 1). Transpiration rate can increase with VPD, potentially increasing calcium access at the growing point and reducing tipburn incidence, but this response varies depending on VPD exposure, as stomata can be closed to prevent waterloss [5, 28]. Tipburn occurrence may have been suppressed at the higher MDTs due to greater VPDs, and therefore affected how MDT may influence tipburn. However, the most likely cause of our high tipburn incidence is a lack of sufficient vertical airflow over the lettuce canopy under rapid growth conditions.

Marketability of crops is influenced by foliage color, with green foliage being undesirable in red-leaf cultivars [30]. Most red, blue, and purple coloration of foliage is primarily caused by anthocyanins [31]. At low temperatures, anthocyanins can accumulate in leaves, inducing darker, more pigmented foliage. The color of ‘Rouxaï RZ’ foliage at an MDT of 26°C was a lighter, more vibrant green than the darker, grayer yellow/red foliage at 20°C, which is consistent with other studies on foliage coloration [17, 32, 33].

Future studies comparing lettuce growth responses to CO_2_ concentrations and temperatures applied at different growth stages are needed to identify when supplemental inputs are most valuable. Esmaili et al. reported lettuce growth responses 10, 20, 30, and 40 d after sowing, with the greatest growth rate change occurring after 30 d [21]. However, this was using constant environmental conditions over the growth cycle, rather than comparing CO_2_ supplementation at different growth stages. Response to MDT may also vary by growth stage; relative growth rate of butterhead lettuce ‘Omega’ 25 d after transplant was greater at 30/25°C than 20/15°C, but by 35 d after transplant, relative growth rate was lowest at 30/25°C [20]. Identifying the growth stage that specific MDT and CO_2_ supplementation is most beneficial enables strategic applications for efficient input use.

In conclusion, under a PPFD of 300 μmol∙m^‒2^∙s^‒1^ and with a 6°C difference in day and night temperature, we recommend growing ‘Rex’ and ‘Rouxaï RZ’ at a CO_2_ concentration of 800 μmol·mol^−1^ and MDT of 23°C as this provided the greatest biomass and leaf number, kept plants moderately compact, and, for ‘Rouxaï RZ’, induced redder foliage than growth at 26°C. Increasing to 1200 a CO_2_ concentration of 800 μmol·mol^−1^ did not provide additional benefits for growth or quality parameters while increasing tipburn occurrence in ‘Rouxaï RZ’.
